# A cross-sectional examination of service complexity in youths with co-occurring autism spectrum disorder and psychiatric or medical diagnoses across service sectors

**DOI:** 10.3389/fpsyg.2022.1027373

**Published:** 2023-02-01

**Authors:** Valbona Semovski, Colin King, Natalia Lapshina, Shannon L. Stewart

**Affiliations:** Faculty of Education, Western University, London, ON, Canada

**Keywords:** autism spectrum disorder (ASD), service complexity, interRAI, youth, co-occurring conditions, medical diagnosis, psychiatric diagnosis

## Abstract

**Introduction:**

Autism spectrum disorder (ASD) is a heterogeneous, life-long, and complex condition. Youth diagnosed with ASD require several supports addressing core symptoms associated with the disorder, but also those resulting from co-occurring mental and physical health conditions. As a result, their care is overseen by numerous professionals spanning various service sectors, but communication between sectors is hindered due to the absence of a standardized assessment system to identify and triage youth to services. A paucity of information surrounding this population’s service use lingers and a siloed delivery system persists.

**Methods:**

Using archival data collected from 1,020 youth between 12 and 18 years of age, this study explored service complexity among autistic youth with and without psychiatric and medical co-occurring conditions in Ontario, Canada. In doing so, a negative binomial regression was utilized to investigate which predisposing, enabling, and need variables were associated with service complexity.

**Results:**

Results revealed that experiencing financial difficulties was not associated with service complexity. However, age, sex, caregiver distress, comorbidity, intellectual disability, and evaluated health status were significant predictors. More specifically, female youth and youth with distressed caregivers had greater mental health service complexity scores. Additionally, youth diagnosed with two or more conditions in addition to ASD who required longer durations of programming, controlling for other predictors, had greater mental health service complexity scores. Yet, youth with an intellectual disability had lower service complexity scores.

**Discussion:**

Clinical implications of this study are discussed to inform future investments into mental health efforts for autistic youth.

## 1. Introduction

Multimorbidity or the co-occurrence of two or more chronic conditions is often the rule, rather than the exception, substantially undermining youth’s functioning (Ferro et al., 2021, p. 105). Youth with multimorbidity are at greater risk of requiring urgent mental health services when compared to children younger than 12 years of age (Semovski et al., 2022) and are more likely to experience greater symptom severity when compared to peers with a singular chronic condition (i.e., medical or psychiatric diagnosis only; Butler et al., 2018, p. 1). When presenting in clinical settings, these youth often exhibit a complex constellation of symptoms and behaviors that require episodic but ongoing care from multi-sectoral services (Lapshina and Stewart, 2019, p. 464; Paton and Hiscock, 2019, p. 2; Stewart et al., 2019, p. 1). This is particularly the case among youth diagnosed with autism spectrum disorder (ASD).

The prevalence of ASD has risen in recent years (Stadnick et al., 2020, p. 2) with an estimated 1 in 66 Canadian children and youth (Autism Ontario, 2020, p. 6) meeting diagnostic criteria. ASD is a heterogeneous, life long and complex condition with nearly half of all autistic individuals having a major co-occurring condition (Cheak-Zamora et al., 2013, p. 448). Autistic youth require several treatment resources for co-occurring medical (e.g., epilepsy, respiratory diseases) and psychiatric diagnoses (e.g., anxiety, depression; Mazurek et al., 2020, p. 401; Brooks et al., 2021, p. 634). The overlap of diagnoses contributes to the complexity of ASD treatment (Stewart et al., 2021a, p. 2) and an increased prevalence of ASD translates into a greater demand for supports (Kogan et al., 2008, p. 1,150; Menezes et al., 2021, p. 2,200) which often exceeds the resources available to youth and their families (Stewart et al., 2021a, p. 2). Despite the gap between service need and corresponding service use, there are a limited number of studies focused on specific factors related to how this population accesses services (Ishler et al., 2022, p. 1,052).

To date, there is limited knowledge about the different types of conditions associated with ASD (Cawthorpe, 2018, p. 2). The differential knowledge about co-occurring conditions among autistic individuals results in barriers to service use. The absence of this information perpetuates a fragmented approach to service delivery among different professionals (Bower et al., 2011, p. 580) due to a lack of multidisciplinary service providers from the onset and heightens the probability of inappropriate referrals (Stewart et al., 2021a, p. 2). This is problematic, considering research has highlighted an association between co-occurrences and mortality among autistic children (Hirvikoski et al., 2016, p. 232). Further, there lacks well-defined treatment guidelines for autistic youth and co-occurring psychiatric and physical health conditions (Stadnick et al., 2020, p. 3), such as attention-deficit/hyperactivity disorder (ADHD), anxiety, mood, sleep disorders and epilepsy which are commonly encountered among autistic individuals (Casanova et al., 2020, p. 1; Bougeard et al., 2021, p. 2). In some instances, having a diagnosis of ASD serves as an exclusionary criterion for mental health services (Mowat et al., 2019, p. 1). This results in unmet health care needs (Mazurek et al., 2020, p. 401; Brooks et al., 2021, p. 634), siloed systems of care (Mowat et al., 2019, p. 1) and poor access to specialized services (Mazurek et al., 2020, p. 3). Autistic individuals have reported poorer quality of health care, the absence of a central, coordinating source of care, and caregivers of youth have indicated they often are disregarded by service providers in decision-making (Menezes et al., 2021, p. 2,200).

The extant literature covering this topic area has generally used small sample sizes, has been void of a conceptual framework guiding analyses, and has focused on psychiatric diagnoses exclusively. On occasion, medical diagnoses are investigated and when doing so, there is concentration on a handful of diagnosis within each study. Thus, information regarding co-occurrences in autistic youth remains in its infancy, but is instrumental in research, education and clinical practice (Cawthorpe, 2018, p. 11). The lack of information available is partially due to the absence of a standardized approach for the provision of pediatric mental health care services in Canada, which is sorely needed. It has been recognized for many years that a more sustainable model of care for complex clients should be implemented that is built on evidence (Navickas et al., 2016, p. 4). Thus, to begin the rehabilitation of the pediatric mental health care system, high quality, comprehensive and standardized data surrounding users that is person-centered and used for multiple applications to support service system integration is needed (Stewart et al., 2022b, p. 3).

There have been several theories advanced to identify predictors of service use, but one of the most widely acknowledged and well-developed is the Behavioral Model of Health Service Use (Andersen, 1968). This model has been used in several studies investigating service use among a variety of client populations (Babitsch et al., 2012, p. 3), including autistic persons (e.g., Paquette-Smith et al., 2019; Platos and Pisula, 2019). Therefore, to begin understanding the nature of service use among autistic youth, this study adopted the Behavioral Model of Health Service Use as an organizing framework. According to the Behavioral Model of Health Service Use or “Andersen’s model,” the probability that youth will enter the mental health care system is determined by contextual and individual factors with population characteristics (i.e., predisposing, enabling and need variables) having received the most support in the literature (Hochhausen et al., 2011, p. 15).

Predisposing factors include individual-level characteristics, such as demographics, biology and social structures that influence health care use (Babitsch et al., 2012, pp. 8–9). As it pertains to child and youth mental health, there have been mixed findings related to the characteristics that differentiate service users from those who are not served. In a systematic review of the literature on Andersen’s model, Babitsch et al. (2012) cited the most frequently researched predisposing variables were age and sex of the individual. As adolescents mature, they are less likely to access mental health services (Cohen and Hesselbart, 1993, p. 52). Further, studies suggest that the annual rates of inpatient, outpatient and residential service use diminishes with increasing 1-year age increments in adolescence (Singh, 2009, p. 387). This pattern has also been corroborated among studies investigating service use (e.g., outpatient, behavioral services) among autistic adolescents and adults (Paquette-Smith et al., 2019, p. 6; Platos and Pisula, 2019, p. 8). The diminished service use with increasing age has been attributed to several beliefs, including reduced motivation on the part of caregivers who may feel a loss of control over older children, a reduction in pressure from schools and teachers prompting caregivers to seek services of older adolescents (Cohen and Hesselbart, 1993, p. 52) and the disproportionate funding available for older, autistic children when compared to their healthcare needs (Kogan et al., 2008, p. 1,153).

The ASD literature has suggested that sex may also alter receipt of mental health services (Ryan et al., 2018, p. 20). Generally, males are more commonly diagnosed with ASD when compared to female counterparts (Ryan et al., 2018, p. 400; Brooks et al., 2021, p. 20). As a result, male youth are commonly referred to treatment (Walrath et al., 2004, p. 298). When accessing treatment, studies have shown that autistic females are more likely to use a wide range of services (e.g., psychiatric and emergency services) when compared to male counterparts. These differences could be due to the type and severity of ASD in females compared to males (Hull et al., 2017, p. 707).

Beyond the individual, there are several factors attributed to the family unit that may promote or hinder help-seeking behaviors. Members of the child or youth’s support network, especially caregivers, are instrumental in obtaining access to mental health services as they are responsible for finding supports, transporting youth to services, and often have jurisdiction to terminate the mental health supports being received (Mayberry and Heflinger, 2013, p. 105). Reardon et al. (2017) conducted a systematic review exploring parent-reported barriers to their child’s access to mental health services and revealed that knowledge and understanding of mental health are facilitators of access to services. Aiding in the understanding of the youth’s mental health difficulties are the caregivers’ own experiences with mental health challenges (Arcia and Fernandez, 2003, p. 164). Further, these experiences are met with heightened stress (Crowell et al., 2019, p. 22) and in turn, may lead to the requirement of respite services to provide relief to the main caregivers (Cooke et al., 2020, p. 2).

Caring for autistic youth poses unique challenges that negatively influences the wellbeing of caregivers (Willet et al., 2018, p. 340). Parents of autistic children report greater feelings of parenting stress than parents of non-autistic youth and when compared to parents of children with other developmental disabilities (McCauley and Solomon, 2022, p. 2). Additionally, they also report more challenges to accessing healthcare services and poorer care quality compared to caregivers of children and youth with other developmental disabilities (Menezes et al., 2021, p. 2,200). This heightened stress may impact the development of ASD and the manifestations of related symptoms and behaviours in children. For instance, parents with greater stress tend to have fewer positive interactions with their children (Giannotti et al., 2021, p. 3) and interactions that lack emotional scaffolding have negative implications on the child’s emotion regulation (Crowell et al., 2019, p. 23). Contrastingly, supportive child-caregiver interactions have been associated with positive adjustment for autistic children (McCauley and Solomon, 2022, p. 3).

Enabling factors involve community or individual-level resources that facilitate access to health care. These factors relate to having appropriate community and individual-level resources necessary to access the care (Andersen, 1995, p. 3). In the pediatric mental health literature, living below the poverty line increases the likelihood that children and youth will receive fewer mental health services and have unmet service needs (Mendenhall, 2012, p. 604). Financial hardships among caregivers of autistic children and youth only amplifies the wedge between them and access to health care services (Karpur et al., 2019, p. 1,654). In an effort to bridge this gap and connect families to a variety of supports, the Government of Ontario in Canada has attempted to address funding issues for ASD services (Mowat et al., 2019, p. 1) with limited success due to the lack of coordination of services, as well as the complex needs of these children (McLaughlin and Schneider, 2019, p. 22).

The need variables in Andersen’s model refer to both perceived and evaluated need; how youth view their own health and functional state or that determined by a health care provider via professional assessments and objective measures, respectively (Andersen, 1995, p. 3). The literature has supported that symptom severity should be associated with the type and level of care received (Mendenhall, 2012, p. 609). For example, caregivers of children and youth with extensive clinical needs, including co-occurring mental health difficulties, use more services than counterparts without (Paquette-Smith et al., 2019, p. 2). Youth with developmental disabilities have a greater risk of mental health or challenging behaviours (Mowat et al., 2019, p. 2). Contributing to some of these challenges for approximately one-third of the population with ASD is the co-occurrence of an intellectual disability (Menezes et al., 2021, p. 2,200). Intellectual disability, characterized by significant limitations in both intellectual functioning and adaptive behaviours (American Psychiatric Association, 2013) negatively impacts cognition, communication and behaviour among autistic individuals (Menezes et al., 2021, p. 2,200). Autistic youth who have an intellectual disability are noted to have received an ASD diagnosis earlier than counterparts with ASD alone, so these youth may receive more diverse services earlier than their peers without a co-occurring intellectual disability (Zablotsky et al., 2015, p. 2).

Though Andersen’s model has been used for decades in health services research, there remain many important gaps in knowledge, especially as it pertains to research among ASD samples. The current study explored predisposing, enabling and need variables related to service complexity among autistic youth in the presence and absence of psychiatric and medical diagnoses. There are two published studies that have investigated service complexity using interRAI data among a pediatric sample. interRAI is a not-for-profit collaborative of over 100 clinicians, researchers and policy experts across the globe with a shared goal of improving quality of life through the development and implementation of comprehensive assessment, screening and care planning tools. The first study investigating service complexity among 330 children with an intellectual disability between 4 and 18 years of age discovered that mental health service use was greater for individuals between 11 and 14 years of age with ASD, a learning or communication disorder, families living with higher family dysfunction and individuals who have experienced interpersonal trauma (e.g., bullying; Stewart et al., 2017). In more recent work, similar themes were highlighted when exploring service complexity among children and youth with an intellectual disorder receiving either inpatient or outpatient services. Main findings suggested that older age, higher family dysfunction, safety risk and cumulative trauma predicted greater service complexity among individuals in outpatient settings, but not inpatient settings (Lapshina and Stewart, 2019, p. 464). Based on the extant literature, it was hypothesized that predisposing, enabling and need variables would be related to service complexity. In particular, it was expected that (i) older youth, (ii) females vs. males, (iii) greater caregiver distress, (iv) financial difficulties, (v) multimorbidity (co-occurring medical and psychiatric diagnoses among youth), (vi) presence of an intellectual disability and (vii) longer durations of care from an agency would be associated with higher levels of service complexity. The identification of significant predictors of service complexity can reveal the characteristics of youth and their families that receive mental health supports. Considering ASD is a lifelong condition, understanding co-occurring conditions and their influence on service complexity is of great importance (Casanova et al., 2020, p. 1). Knowledge about ASD, especially in the context of co-occurring diagnoses is instrumental in creating policies that would guide investments into prevention and intervention efforts.

## 2. Materials and methods

### 2.1. Participants and procedure

The de-identified archival data for this non-experimental, cross-sectional study was obtained from 1,020 youth (*M*_*age*_ = 14.5, *SD*_*age*_ = 1.8) between 12 and 18 years of age who identified as a cisgender male or female. To be included in the study, youth must have been diagnosed by a psychiatrist, attending physician or qualified psychologist with ASD based on diagnoses obtained from major diagnostic guides (i.e., The Diagnostic and Statistical Manual of Mental Disorders and the International Classification of Diseases). In this particular study, less than 5 youth per cell size identified as transgender and/or non-conforming and thus, were excluded from analysis. Information from youth was collected by trained assessors at participating community mental health agencies in Ontario, Canada between June 2012 and November 2020. Assessors include professionals from a variety of backgrounds (e.g., social work, child and youth work, nursing) with at least 2 years of experience with this age group. Prior to administering interRAI instruments in their practice, assessors receive a 2-day training program, which includes a competency evaluation to qualify them to administer and interpret the respective interRAI instruments.

At the community mental health agencies, interRAI instruments are integrated into their standard of care. Thus, caregivers or the youth themselves are asked for permission to have their de-identified data included in interRAI’s secured repository upon starting clinical services in these settings. After obtaining informed consent from the caregiver(s) or the youth themselves, the assessors administer a semi-structured interview with the youth, guardians, teachers and where appropriate, other professionals to gain information about the youth. Additionally, assessors can supplement information obtained from their clinical interviews by reviewing the youth’s medical records, academic assessments or other relevant and accessible information. Once information is collected, it is stored in a de-identified web-based software system that securely stores the data on the interRAI Canada server and is housed at a partner university. The system randomly generates a unique participant number for each youth. Research Ethics Board approval was granted for the secondary analysis of the data.

As shown in Table 1, most youth were male (75.4%) and approximately a quarter (24.6%) were reported to have an intellectual disability. From each respective group, 4.90, 15.5, and 4.22% of autistic youth in groups 1, 2, and 3 had a co-occurring intellectual disability. The majority of the study sample (84.6%) had ASD and an additional diagnosis. Several youth had one psychiatric diagnosis (29.2%) in addition to ASD, but majority of the study sample (53.1%) had several co-occurring psychiatric diagnoses. The most common psychiatric diagnoses in this sample were ADHD followed by anxiety disorders with 48.9 and 42.9%. Approximately 83.4% of youth with ADHD belonged to group 2 and 16.6% to group 3. Additionally, 84.7% of youth with an anxiety disorder comprised group 2 and 15.3% were in group 3. Approximately 16.6% of the sample had a medical diagnosis in addition to ASD. The most common medical diagnosis was asthma with 7.9% of the sample reporting the diagnosis; 13.6% of these youth were in group 2 and 86.4% in group 3. This was followed by epilepsy which was endorsed by 4.8% of the overall sample; 22.5% in group 2 and 77.5% in group 3. Further, the majority of the study sample were in the legal guardianship of both parents (61.6%), did not experience financial difficulties (96.1%), but endorsed some level of caregiver distress (55.9%).

**TABLE 1:** Sample characteristics (*N* = 1,020).

	*n* or *M*	% or *SD*
**Sex**		
Female	251	24.6%
Male	769	75.4%
**Age**	14.5	1.8
Legal guardianship		
Both parents	628	61.6%
Mother only	277	27.2%
Father only	45	4.4%
Neither parent but other relative(s) or non-relative(s)	30	2.9%
Child protection agency	38	3.7%
Other^a^	2	0.20%
**Caregiver distress**		
None	449	44%
Mild	346	33.9%
Moderate	196	19.2%
Severe	29	2.8%
**Financial difficulties**		
Yes	40	3.9%
No	980	96.1%
**Comorbidity**		
Group 1: ASD	157	15.4%
Group 2: ASD + a psychiatric diagnosis or a medical diagnosis	718	70.4%
Group 3: ASD + co-occurring psychiatric and medical diagnoses	145	14.2%
**Psychiatric diagnosis**		
Reactive attachment disorder	17	1.67%
Attention-deficit/hyperactivity disorder	499	48.9%
Disruptive behaviour disorder	199	19.5%
Learning or communication disorder	380	37.3%
Substance-related disorder	22	2.2%
Schizophrenia and other psychotic disorders	13	1.3%
Mood disorders	154	15.1%
Anxiety disorders	438	42.9%
Eating disorders	11	1.1%
Sleep disorders	19	1.9%
Adjustment disorders	34	3.3%
**Medical diagnosis**		
Asthma	81	7.9%
Diabetes mellitus	13	1.3%
Epilepsy or seizure disorder	49	4.8%
Fetal alcohol effects/syndrome	30	2.9%
Migraine	23	2.3%
Traumatic brain injury	6	0.6%
**Intellectual disability^b^**		
Yes	251	24.6%
No	769	75.4%
**Evaluated health status**		
<90 days	295	28.9%
91 or more days	725	71.1%

^a^Due to ethical considerations, select legal guardianship categories could not be reported and were collapsed into “Other.” ^b^Intellectual disability is defined as an Intelligent Quotient (IQ) of less than 70.

### 2.2. Measures

#### 2.2.1. InterRAI child and youth mental health instrument

The interRAI ChYMH (Stewart et al., 2015) is a need-based assessment used in mental health settings at admission, discharge, every 90 days in the event of longer client stays, and if there is a significant change in a youth’s care planning needs. For the purposes of the current study, all information analyzed was reflective of initial admission. The clinician-administered instrument is appropriate for use with children between 4 and 18 years of age and supports a variety of applications including outcome measurement, resource allocation and comprehensive care planning in several domains of the youth’s life (e.g., traumatic life events, education). Further, the instrument contains over 400 evidence-based items that generate scales and algorithms capturing this information and utilizes specific look-back periods to ensure that the information gathered is a reliable measure of clinical characteristics that reflect the youth’s current strengths and needs (Stewart et al., 2022b, p. 5). The assessment takes approximately 45–90 min to complete, depending on case complexity. The instruments contained within the interRAI Child and Youth suite have been developed to provide an integrated health information assessment system with multiple applications (Hirdes et al., 2020, p. 14; Stewart et al., 2022b, p. 15).

#### 2.2.2. InterRAI child and youth mental health for developmental disabilities instrument

Among the instruments developed by interRAI for use with children and youth is the interRAI ChYMH-DD (Stewart et al., 2015) that is intended for use with individuals between 4 and 21 years of age with developmental disabilities. Similar to the ChYMH, the ChYMH-DD contains similar scales and algorithms, specific observation periods and approximate time for completion. The ChYMH-DD obtains information regarding care planning in domains that may be relevant to this demographic (e.g., accessibility and mobility, modified nutrition intake). Further, both the ChYMH and ChYMH-DD contain scales and algorithms that have demonstrated strong reliability and validity across multiple contexts (Stewart et al., 2015, 2019, 2020a,b,c, 2021b, 2022a; Stewart and Hamza, 2017; Lau et al., 2018, 2019, 2021; Hirdes et al., 2020; Redquest et al., 2020; Stewart and Babcock, 2020; Barbaree et al., 2021; Li et al., 2021).

#### 2.2.3. Predisposing variables

In the current study, demographic information such as age and sex were gathered from the youth and represented predisposing variables. Youth’s sex was coded as *male* or *female*. Age was a continuous variable ranging from 12 to 18 years.

##### 2.2.3.1. Caregiver distress

The caregiver distress composite variable measures the degree and diversity of caregiver distress factors. It consists of three items (i.e., parent/primary guardian had experienced major life stressors in the last 90 days; parent/primary guardian was unable or unwilling to continue in caring activities; parent/primary guardian expresses feelings of distress, anger or depression) which are dichotomized to reflect the endorsement or absence of caregiver distress. Responses were summed, resulting in a score ranging from 0 to 3 with higher scores indicative of greater caregiver distress. As a naming convention for this study, scores of 1 indicate *mild* caregiver distress, scores of 2 indicate *moderate* caregiver distress and a score of 3 indicates *severe* caregiver distress.

#### 2.2.4. Enabling variable

Caregivers of youth or the youth themselves were asked if in the last 30 days, due to limited funds, were they required to make trade-offs among purchasing any of the following: adequate food, shelter, clothing, prescribed medications, sufficient home heat or cooling, or necessary health care. Response options included *yes* or *no*. In this study, “trade-offs” refer to situations resulting from budget constraints that involve the sacrifice of something in return for a gain. The ChYMH and ChYMH-DD do not collect information on specific income levels. Consequently, a proxy for financial problems (i.e., financial trade-offs) represented financial difficulties in the current study.

#### 2.2.5. Need variables

##### 2.2.5.1. Comorbidity

The ChYMH and ChYMH-DD obtain diagnostic and other health information pertaining to the youth. For psychiatric disorders, clinicians were asked to identify all provisional diagnostic categories determined by a psychiatrist, physician or psychologist and rank their importance as factors contributing to the youth’s admission. The most prevalent diagnoses included reactive attachment disorder; attention-deficit/hyperactivity disorder; learning or communication disorder; substance-related disorders; schizophrenia and other psychotic disorders; mood disorders; anxiety disorders; eating disorders; sleep disorders and adjustment disorders. Response options on the interRAI instruments include not present; most important; second most important; third most important; less important and no provisional diagnosis. For the purposes of this study, response options were dichotomized to indicate either the presence or absence of a specific diagnosis.

As it pertains to medical diagnoses, clinicians documented the presence of medical diseases or infections relevant to the youth’s status either through reviewing clinical records and/or speaking to the caregiver(s) and youth. Medical diagnoses most commonly captured on the interRAI instruments include asthma; diabetes mellitus; epilepsy or seizure disorders; fetal alcohol effects/syndrome; migraine and traumatic brain injury. Response options are not present, primary diagnosis/diagnoses for current stay, diagnosis present, receiving active treatment and diagnosis present, monitored but no active treatment. Similarly, to psychiatric diagnoses, medical diagnoses were dichotomized to illustrate the presence or absence of a specific diagnosis.

Therefore, youth who received only a diagnosis of ASD, in the absence of any psychiatric or medical diagnosis comprised *group 1* in the current study. *Group 2* represented autistic youth with either one psychiatric or medical diagnosis. Finally, *group 3* consisted of autistic youth with two or more psychiatric and medical diagnoses.

##### 2.2.5.2. Intellectual disability

Clinicians documented the youth’s intellectual functioning if available from prior testing. Response options included *very superior* (IQ > 130), *superior* (IQ of 120–130), *average* (IQ of 90–120), *low average* (IQ of 80–89), *borderline range* (IQ of 70–79), and *extremely low range* (IQ < 70). Consistent with thresholds published in the literature, youth who had intellectual functioning less than 70 were considered to have an intellectual disability (Alvares et al., 2020, p. 220). Numerous articles have investigated the relationship between intellectual disability and ASD among pediatric samples. Intellectual disability also has significant overlap with several other psychiatric diagnoses. The focus for this paper was other psychiatric and medical diagnoses that are least commonly investigated in the literature. The decision to have intellectual disability separately, rather than collapsing it with the other psychiatric diagnoses was done in an effort to speak to the unique relationship between an ID diagnosis and service complexity among this sample of autistic youth, separate from the discussion around other psychiatric and medical co-occurrences. This is aligned with recent scholarly works that have recommended researchers examine ID independent of other co-occurring psychiatric diagnoses (Ishler et al., 2022, p. 1,062). Therefore, intellectual disability in the current study is a dichotomous variable coded as 0 (IQs above 70) or 1 (IQs below 70); youth with IQs above 70 were collapsed into a group and compared to youth with IQs below 70.

##### 2.2.5.3. Evaluated health status

This variable captures the estimated number of days the youth is expected to remain in the program being offered upon admission, from the time of assessment to the time of discharge. Response options for evaluated health status are 1–7, 8–14, 15–30, 31–90, and 91 or more days. Responses were dichotomized to illustrate *shorter (1–90 day)* and *longer (91 or more days)* stays, coded as 0 and 1, respectively.

#### 2.2.6. Service complexity

The service complexity variable captures a variety of services and formal care providers spanning multiple service sectors and disciplines (e.g., mental health agencies, schools) while delineating the intensity and nature of the service needs. More complex youth receive a higher number of the services (Stewart et al., 2017). More specifically, a score of one is given for services obtained from each of the following formal care providers in the last 90 days: psychiatrist, social worker, psychologist or psychometrist, child protection and case management; with scores ranging from 0 to 5. Any youth who received prior inpatient mental health admissions also receive a score of one. With respect to specific services that were scheduled or received in the last 30 days, a score of one is given if the youth received 3 or more of the following interventions: life skills training, social skills, family functioning, anger management, behaviour management, crisis intervention, family preservations, family support and medication management. Lastly, a score of one is given if the youth received 2 or more of the following in the last 90 days: acute hospital admissions, emergency department visits or physician visits. This results in a composite variable with values ranging from 0 to 8, where higher scores indicate more complex services received by the youth and their families.

### 2.3. Analytic strategy

The data were initially screened to determine suitability for analyses. Due to the skewed nature and count characteristic of the dependent variable in the current study, two types of distributions are appropriate for this type of data. The first is a Poisson regression which is appropriate for count data with equidispersion, or a distribution where the conditional mean and variance of the dependent variable are equal. If equidispersion is absent and the variance is larger than the mean, then the data is said to be overdispersed and a negative binomial regression is more appropriate (Nussbaum et al., 2008). In the current study, overdispersion was detected through a test of the dispersion parameter which confirmed that a negative binomial model fit the data better than a Poisson distribution.

Negative binomial regression was used to examine whether the dependent variable, service complexity among autistic youth was associated with independent variables such as the youth’s age, sex, caregiver distress, financial difficulties, co-occurring conditions, presence of an intellectual disability and evaluated health status spanning June 2012 and November 2020. Summary statistics for demographic information were examined. Correlations (Pearson’s *r*) along with independent sample *t*-tests and ANOVAs were carried out to determine the degree to which all variables were associated with each other. Following this, univariate (unadjusted) and multivariate (adjusted) models were computed investigating the effects of the independent variables on service complexity.

In conducting the regression, each independent variable was entered in the model hierarchically using recommendations set out in Mendenhall (2012); predisposing variables first, followed by enabling variables and finally, need variables. Improvements to model fit were determined by consulting Pearson’s chi-square values, the Alkaike Information Criterion (AIC) and Bayesian Information Criterion (BIC) (Nussbaum et al., 2008). Smaller AIC/BIC values were indicative of a better fitting model (Hayat and Higgins, 2014). Adjusted incidence rate ratios (IRR) were calculated using the multivariate negative binomial regression. Adjusted IRR were calculated by exponentiating the regression coefficients, estimating the ratio by which service complexity changes for each independent variable examined. Reference categories were males, no caregiver distress, no financial difficulties, ASD only, presence of an intellectual disability and 91 or more days of programming. All statistical analyses were conducted using SAS 9.4 (SAS Institute, Cary NC).

## 3. Results

### 3.1. Descriptive data for services utilized

In the study sample, 36 youth (3.53%) did not receive any type of formal care for their psychiatric or medical needs in the last 3 years. The average number of services accessed by autistic youth was 3.47 (*SD* = 1.89). Approximately half (48.3%) of the youth received four or more services. The most common services accessed were formal care by a psychiatrist (66.5%), social worker (65.7%), case manager (55.5%) and physician (51.1%). Additionally, very few youth (*n* < 5) reported receiving 9 services (i.e., formal care from psychiatry; social work; psychology, psychometry, psychology associate; child protection; case management; inpatient mental health admission; acute hospital admission; emergency department visit; and physician visit).

The focus of intervention varied across the study sample. The most common issues that were investigated in the previous 30 days were social skills (36.7%), behaviour management (35.8%) and medication management (27.3%). The least common focus for intervention among autistic youth was family preservation (11.2%). Table 2 summarizes the formal care received and the focus of intervention for each respective group in the study. Group 1 are youth with a sole diagnosis of ASD, group 2 is comprised of autistic youth with either one psychiatric diagnosis *or* one medical diagnosis and group 3 consists of autistic youth with at least one psychiatric disorder *and* one medical diagnosis. The average service complexity score among this sample was 2.51 (*SD* = 1.78).

**TABLE 2:** Services utilized by comorbidity group.

	Group 1		Group 2		Group 3	
	*n*	%	*n*	%	*n*	%
**Formal care**						
Psychiatrist^a^	62	10.3%	447	74.0%	95	15.7%
Social worker^a^	71	11.9%	445	74.5%	81	13.6%
Psychologist, psychometrist, psychological associate^a^	44	9.6%	346	75.6%	68	14.9%
Child protection^a^	37	13.0%	201	70.5%	47	16.5%
Case management	70	12.4%	405	71.6%	91	16.1%
Inpatient mental health admissions	27	9.0%	212	70.9%	60	20.0%
Acute hospital admission (non-psychiatric)	5	9.6%	38	73.1%	9	17.3%
Emergency department visit	18	11.4%	116	73.9%	23	14.7%
Physician visit	80	15.4%	348	66.8%	93	17.8%
**Focus of intervention**						
Life skills training	30	12.5%	166	69.5%	43	18.0%
Social skills	46	12.3%	269	71.9%	59	15.8%
Family functioning	32	12.0%	196	74.0%	37	14.0%
Anger management^b^	19	10.2%	144	77.0%	24	12.8%
Behaviour management	46	12.6%	261	71.5%	58	15.9%
Crisis intervention	19	11.2%	123	72.4%	28	16.4%
Family preservation	15	13.2%	83	72.8%	16	14.0%
Family support	24	11.9%	143	71.1%	34	16.9%
Medication management	30	10.8%	194	69.8%	54	19.4%

^a^111 missing observations. ^b^251 missing observations. Group 1, sole diagnosis of ASD; Group 2, ASD with either one psychiatric or medical diagnosis and; Group 3, ASD with two or more psychiatric/medical diagnoses.

### 3.2. Bivariate analyses

In the present study, correlations (Pearson’s *r*) along with independent sample *t*-tests and ANOVAs were carried out to determine the degree to which all variables were associated with each other. Correlational results indicated that caregiver distress and financial difficulties [χ^2^ (3) = 39.99 *p* < 0.0001]; caregiver distress and co-occurring conditions [χ^2^ (6) = 17.13 *p* = 0.0088]; caregiver distress and evaluated health status [χ^2^ (3) = 9.16 *p* = 0.0273]; and co-occurring conditions and intellectual disability [χ^2^ (2) = 9.05 *p* = 0.0109] were associated with each other. Additionally, an independent sample *t*-test between sex and age [*t* (426) = –290, *p* = 0.0039] revealed that they were correlated with each other. The bivariate correlations between all other independent variables were non-significant.

### 3.3. Predictors of service complexity

A number of variables examined were significantly associated with service complexity at the bivariate level. At the bivariate level, age (*p* = 0.0013), sex (*p* = 0.0048), co-occurring conditions (*p* < 0.0001), intellectual disability (*p* = 0.0003), mild caregiver distress (*p* < 0.0001), moderate caregiver distress (*p* < 0.0001), severe caregiver distress (*p* = 0.0049) and evaluated health status (*p* < 0.0001) were significant predictors of service complexity. Financial difficulties (*p* = 0.265) was a non-significant predictor of service complexity.

Although most of the variables were significantly associated with service complexity at the bivariate level, the results did not control for the effect of other predictors. Therefore, the net effect of each variable on service complexity could not be obtained. Thus, a hierarchical regression model was conducted to predict service complexity based on the youth’s age, sex, caregiver’s distress, financial difficulties, co-occurring conditions, intellectual disability status and evaluated health status. This multivariate model adjusted for the effect of all other predictors. Table 3 summarizes the statistical output for the comparison of the respective negative binomial models, highlighting the model with all predictors being superior to the constant-only model and relative to the model with fewer independent variables.

**TABLE 3:** Model fit statistics summary for hierarchical negative binomial regression model.

Step	*X* ^2^	*P*	AIC	BIC
1. Constant-only	990.07^a^	0.736	3950.04	3959.90
Step 1 + age	997.07^b^	0.675	3941.80	3956.59
Step 2 + sex	1002.44^c^	0.622	3937.38	3957.09
Step 3 + caregiver distress	1049.93^d^	0.211	3866.66	3901.15
Step 4 + financial difficulties	1049.73^e^	0.216	3866.66	3901.15
Step 5 + co-occurrences	1056.09^f^	0.158	3841.57	3890.85
Step 6 + intellectual disability	1059.29^g^	0.137	3831.35	3885.56
Step 7 + evaluated health status	1067.89^h^	0.009	3817.62	3876.75

^a^*df* = 1,019, ^b^*df* = 1,018, ^c^*df* = 1,017, ^d^*df* = 1,014, ^e^*df* = 1,013, ^f^*df* = 1,011, ^g^*df* = 1,010, ^h^*df* = 1,009.

The results of the negative binomial regression (Table 4) conducted on mental health service complexity indicated that age was a significant predictor (IRR = 1.03; 95% CI = 1.01–1.06; *p* = 0.0102). Specifically, for an increase of 1 year in age, the service complexity score was 1.03 times greater for youth. Similarly, the youth’s sex was a significant predictor of service complexity (IRR = 1.14; 95% CI = 1.04–1.25; *p* = 0.0061), such that the risk of higher service complexity is 1.14 times greater for females when compared to male peers. Controlling for all other predictors, caregiver distress significantly predicted mental health service complexity. The service complexity score was 1.23 times greater for youth with mild caregiver distress (IRR = 1.23; 95% CI = 1.12–1.36; *p* < 0.0001), 1.58 times greater for youth with moderate caregiver distress (IRR = 1.58; 95% CI = 1.41–1.75, *p* < 0.0001) and 1.38 times greater for youth with severe caregiver distress (IRR = 1.38; 95% CI = 1.07–1.74, *p* = 0.0096) when compared to youth reporting no caregiver distress.

**TABLE 4:** Negative binomial regression predicting mental health service complexity.

Variable	*B*	Standard error	Wald chi-square	*p*	IRR	95% CI
**Constant**	–0.11	0.19	0.34	0.5579	0.90	[0.63, 1.29]
**Predisposing variables**						
Age	0.03	0.01	6.60	0.0102	1.03	[1.01, 1.06]
**Sex**						
Female	0.13	0.05	7.53	0.0061	1.14	[1.04, 1.25]
Male (RC)						
**Caregiver distress**						
None (RC)						
Mild	0.21	0.05	17.59	<0.0001	1.23	[1.12, 1.36]
Moderate	0.45	0.06	68.80	<0.0001	1.58	[1.41, 1.75]
Severe	0.32	0.12	6.71	0.0096	1.38	[1.07, 1.74]
**Enabling variables**						
Financial difficulties						
Yes	–0.02	0.10	0.03	0.8589	0.98	[0.80, 1.20]
No (RC)						
**Need variables**						
Co-occurrences						
Group 1: ASD (RC)						
Group 2: ASD + a psychiatric diagnosis or a medical diagnosis	0.30	0.07	20.65	<0.0001	1.36	[1.19, 1.55]
Group 3: ASD + co-occurring psychiatric and medical diagnoses	0.41	0.08	25.99	<0.0001	1.51	[1.29, 1.77]
**Intellectual disability**						
Yes (RC)						
No	0.18	0.05	12.07	0.0005	1.19	[1.08, 1.32]
**Evaluated health status**						
1–90 days	–0.19	0.05	15.42	<0.0001	0.83	[0.75, 0.91]
91 or more days (RC)						

RC, reference category; IRR, incidence rate ratio; CI, confidence interval.

Experiencing financial difficulties was not a significant predictor of mental health service complexity (IRR = 0.98; 95% CI = 0.80–1.20, *p* = 0.86). When compared to youth with a sole diagnosis of ASD, youth with at least one additional psychiatric or medical diagnosis are predicted to have a higher service complexity score. More specifically, the predicted service complexity score increases by 35.6% for youth in group 2 when compared to their peers in group 1 (IRR = 1.36; 95% CI = 1.19–1.55, *p* < 0.0001). Similarly, the predicted service complexity score increases by approximately 51% for youth in group 3 when compared to their peers with a sole diagnosis of ASD (IRR = 1.51; 95% CI = 1.29–1.77, *p* < 0.0001). Further, those without an intellectual disability are expected to have a complexity score 1.19 times greater than peers with an intellectual disability (IRR = 1.19; 95% CI = 1.08–1.32, *p* = 0.0005). Finally, youth expected to remain in programming for 1–90 days relative to youth who have longer programming needs are expected to have a service complexity score 0.83 times smaller (IRR = 0.83; 95% CI = 0.75–0.91, *p* < 0.0001).

## 4. Discussion

The current study examined service complexity among autistic youth, as reported by youth or their caregivers. Autistic youth require several treatment resources for co-occurring medical (e.g., epilepsy, respiratory diseases) and psychiatric diagnoses (e.g., anxiety, depression; Mazurek et al., 2020, p. 401 Brooks et al., 2021, p. 634) and the overlap of diagnoses contributes to the complexity of ASD treatment (Stewart et al., 2021a, p. 2). However, there are a limited number of studies focused on specific factors related to service use (Ishler et al., 2022, p. 1,052). This study addresses several limitations identified in the existing literature by utilizing a large sample size, adopting a conceptual framework to guide the analyses and focuses on both medical and psychiatric diagnoses. The study hypothesis was partially supported as youth’s age, sex, caregiver distress, co-occurrences, presence of an intellectual disability and evaluated health status were significant predictors of service complexity. However, financial difficulties were not.

This research study found an increase in service complexity with every 1-year increment in age. Overall, Cidav et al. (2013) found a similar trend when investigating age-related variation in health service use. In their study, total expenditures increased by 5% with each year of age. More specifically, they found that as their participants got older, the use of expenditures and long-term care, psychiatric medications, case management, medication management, day hospitalizations and respite services increased. This association between age and select service use can partially be described by the symptom severity among autistic youth. Despite several studies supporting a general improvement of ASD symptoms as individuals age (Woodman et al., 2015, p. 2), there are a vast number of individuals who do not experience such improvements and some may even experience the worsening of symptoms, especially in the presence of co-occurring conditions. Adolescence coincides with the onset of chronic mental health problems (Ryan et al., 2018, p. 25) which may prompt or increase mental health service use. Thus, the increase in service complexity as youth age may be representative of an appropriate service response to these needs in the community.

The heightened service complexity score as youth age can also be reflective of the avenues in which this age group receive services for their challenges. In Ontario, autistic youth are eligible to receive services through their local school boards, in addition to mental health settings (Paquette-Smith et al., 2019, p. 7). These settings for service provision are captured using the interRAI ChYMH and may be reflected in the results of this study. Although time of diagnosis was not investigated in this study, our study sample may also be comprised of youth who have received a diagnosis of ASD later in life. The literature suggests that a late diagnosis of ASD is associated with a more pronounced set of symptoms and challenges (Cidav et al., 2013, p. 7) and these youth receive other diagnoses before receiving an ASD diagnosis (Miodovnik et al., 2015, p. 1). Alternatively, these results can also be attributed to a cohort effect; as youth age, they are made aware and access more services available to them.

In this study, females with ASD when compared to males with ASD were predicted to have a higher service complexity score. The study’s findings are aligned with research suggesting that sex differences alter the receipt of mental health resources (Ryan et al., 2018, p. 20). When accessing treatment, autistic females are more likely to use a range of services when compared to autistic male counterparts. These differences could be due to the type and severity of ASD in females (Hull et al., 2017, p. 707) and are mediated by many biological and cognitive causes (Lai et al., 2011, p. 2). The literature highlights evidence of differing clinical presentations of ASD traits in females when compared to males, with females often exhibiting greater severity in symptoms and co-occurring conditions, such as an intellectual disability. Additionally, females are thought to be more socially competent and more successful at masking ASD symptoms related to social impairments, resulting in misdiagnosis, delayed diagnosis or in some cases, not being recognized at all (Tubío-Fungueiriño et al., 2021, p. 2,191). This delayed access to services is a disadvantage for females as it lends to interventions that are not timely and possibly an exacerbation of maladaptive behaviours (Tubío-Fungueiriño et al., 2021, p. 2,197).

Regardless of the youth’s sex, the constellation of symptoms characteristic of ASD can be distressing for caregivers. Caring for autistic youth is complex, accompanied by a heightened economic and social cost to caregivers (Cooke et al., 2020, p. 2). An estimated 85% of autistic individuals present with limitations cognitively or adaptively, increasing the probability that long-term assistance is required across the lifespan (Karst and Van Hecke, 2012, p. 248). Parents of autistic children report greater feelings of parenting stress than parents of non-autistic youth and when compared to parents of children with other developmental disabilities (McCauley and Solomon, 2022, p. 2). Families affected by ASD are also faced with navigating an uncoordinated system where there are often delays in access to treatment (Karst and Van Hecke, 2012, p. 248).

Additionally, studies have suggested the development of ASD and the manifestation of related symptoms and behaviours in youth may be affected by interactions initiated by caregivers. For instance, parents with greater stress tend to be less sensitive and more directive in interactions with their children. These interactions that lack emotional scaffolding have negative implications on the child’s emotion regulation (Crowell et al., 2019, p. 23). This association between caregiver distress and service complexity was highlighted in the present study. Youth who reported caregiver distress were predicted to have higher service complexity scores when compared to peers with no caregiver distress. The study results lend support to the assertion in the literature that caregivers who recognize their youth’s problems and view them negatively are more likely to seek help and access mental health services relative to those who do not perceive the diagnosis to have negatively impacted their family dynamic (Reardon et al., 2017, p. 624). Future research could expand on these findings and investigate different aspects of caregiving, including positive influences of parenting an autistic child (e.g., levels of empathy, caregivers’ communication skills) to further inform the intervention process and effective supports (Samadi and Samadi, 2020, p. 7).

In conjunction with the emotional cost of caring for autistic youth, the commitment to supporting their child’s prognosis may increase financial burden for caregivers. These demands include, but are not limited to, a greater investment in their child’s education and medical care and fewer opportunities for work and leisure (Karst and Van Hecke, 2012, p. 254). In the present study, financial difficulties were not a statistically significant predictor of service complexity. However, youth reporting financial problems experienced a decrease in their predicted service complexity score when compared to peers with no financial difficulties, insinuating a diminished access to and use of services. This finding is aligned with the extant literature suggesting financial hardships may be a barrier to ASD services for families (Karst and Van Hecke, 2012, p. 7; Cooke et al., 2020, p. 255).

Individuals with developmental disabilities have a higher risk of developing mental health difficulties and in turn, incur a greater risk of physical health problems as well (Mowat et al., 2019, p. 2). It is estimated that about half of all autistic individuals having a major co-occurring condition (Cheak-Zamora et al., 2013, p. 448). A recent community study suggested rates much higher, citing that 92% of autistic youth receiving mental health services met criteria for at least one additional psychiatric diagnosis (Brookman-Frazee et al., 2018, p. 14). Autistic individuals are thought to be at risk for higher prevalence of common psychiatric diagnoses compared to non-autistic individuals (Ivanovic, 2021, p. 1) and youth with multimorbidity are more likely to experience greater symptom severity when compared to peers with a singular chronic condition (Butler et al., 2018, p. 1). As a result of several co-occurrences, they require ongoing care from multi-sectoral services (Lapshina and Stewart, 2019, p. 464). Our study endorsed that youth with co-occurring diagnoses would experience an increase in service complexity scores. The greatest increase in service complexity was exhibited by the group characterized by co-occurring ASD, psychiatric and medical diagnoses.

This finding is similar to Brooks et al. (2021) that found individuals with chronic medical and psychiatric diagnoses had increased interactions with the health care system. It also aligns with previous work that has identified an increased dependence on the healthcare system with an increase in co-occurring conditions among autistic individuals (Schlenz et al., 2015, p. 2; Cummings et al., 2016, p. 2,382). For example, co-occurring medical and psychiatric diagnoses result in higher levels of morbidity, depression and an overall reduction in wellbeing. This contributes to frequent visits to professionals across disciplines and across the health care system (Casanova et al., 2020, p. 1). Associated with this finding is the youth’s evaluated health status. In this study, youth who required shorter durations of programming from agencies were predicted to have a lower service complexity score when compared to peers requiring longer durations of programming.

Among these co-occurrences is the presence of an intellectual disability. The co-occurrence of an intellectual disability with ASD is estimated to be as high as 50–70% (Stewart et al., 2021a, p. 2) and generally, individuals with this co-occurrence report worse outcomes. In contrast to prior studies, the results herein did not provide support for the hypothesized association between intellectual disability and overall service complexity in autistic youth. The study results suggested that youth with ASD and an intellectual disability experienced an overall decrease in their service complexity score. Typically, comorbid intellectual disability adds cognitive, communication and behavioral impairments to the core presentation of ASD (Menezes et al., 2021, p. 2,200) and there is clinical heterogeneity in both intellectual disabilities and ASD. The burden of the co-occurrence occurs at earlier ages and results in inequalities regarding receipt of services (Casanova et al., 2020, p. 5). Several professionals working with individuals diagnosed with an intellectual disability report a lack of confidence in managing challenging behaviors that often accompany such client profiles (Ong et al., 2017, p. 295). This translates into greater unmet health care needs among children with co-occurring ASD and intellectual disability (Menezes et al., 2021, p. 2,200). Autistic youth with a co-occurring intellectual disability have unmet needs at greater frequencies than youth with only ASD (Zablotsky et al., 2015, p. 7). Our study’s findings may further elucidate the discrepancy between service use and need among youth diagnosed with ASD and an intellectual disability.

### 4.1. Clinical implications

The findings of this study present a number of implications for mental health professionals and researchers working with autistic youth and their families. The importance of predisposing, enabling and need variables highlight the necessity to assess and monitor a variety of facets of the youth’s life using a comprehensive, standardized assessment system to support comprehensive assessments, individualize care planning, resource allocation and quality assurance. In the present study, 15% of the sample met diagnostic criteria for only ASD, with majority having co-occurring diagnoses. Youth who experience co-occurring diagnoses experience the mental health service system differently and are predicted to have greater service complexity. The study’s findings highlight the utility of thinking differently for youth with a sole diagnosis of ASD and those with a more diverse constellation of symptoms due to co-occurrences across the lifespan. The co-occurring conditions introduce heterogeneity to the ASD presentation, underscoring the importance of tailored approaches to case conceptualization and support. Assessments within clinical practice should focus on core ASD symptoms, but also on a wider range of psychiatric complaints, including somatic symptoms and where appropriate, physical exams (Bougeard et al., 2021, p. 14). In doing so, comprehensive tools such as the interRAI ChYMH-ChYMH-DD or its equivalent should be adopted into practice.

Despite greater contact with service care providers among youth with multimorbidity, individuals with co-occurring conditions experience more difficulties with fragmentation of care since most supports are often focused on one diagnosis (Barnett et al., 2012, p. 41). Management of symptoms and behaviors evident in clinical settings should be met with a multidisciplinary team approach comprised of several professionals, as these youth consult several formal care providers for their needs. Female youth in particular may be at risk for more frequent and intensive services, as was highlighted by an increase in service complexity relative to male peers. Professionals working with autistic, female youth should be mindful of sex differences when conducting assessments and interventions in attempts to curtail detrimental developmental trajectories and minimize the likelihood female youth will require intensive programming for their needs. Further, it has been suggested that each client have a dedicated clinician that would spearhead the youth’s care coordination across service sectors (Barnett et al., 2012, p. 41), in an effort to minimize fragmented care for individuals with co-occurrences. The prevalence of youth with co-occurring ASD, psychiatric and/or medical diagnoses should be incorporated into clinical guidelines for assessing ASD. Most professional guidelines are created for single diagnoses (Barnett et al., 2012, p. 41). Conversely, interRAI care planning protocols, or Collaborative Action Plans (CAPs), utilize a case-finding methodology that is client-centred, wholisitc and focus on clinical needs, preferences and strengths of the child, youth, and their family (Stewart et al., 2015) within the context of the service system. Further research in the area of multimorbidity among autistic youth should be conducted to obtain evidence-based information and provide strong professional guidelines and recommendations outlining care pathways for this demographic.

Although financial difficulties in this study were not predictive of service complexity, this was likely due to the way financial difficulties were captured and the composition of the study sample (e.g., older with majority absent of an intellectual disability). Younger children and/or more complex presentations of ASD (e.g., co-occurring intellectual disability) may play a decisive role in the amounts and types of services used, so personal income should be considered. Financial resources may be particularly important for those families with youth with very complex needs (Willet et al., 2018, p. 349). If practicing in locations where supplemental funding is not provided by local governments or agencies, clinicians should devise a range of appropriate options and supports to address the needs of their caseload. If possible, the services offered should be delivered in coordination with all members of the youth’s extended healthcare team, including their caregivers.

Caregiver distress resulted in an increase in service complexity underscoring the importance of involving caregivers in interventions related to ASD and assessing and monitoring caregivers’ perceptions of their youth’s overall health journey. Interventions provided to youth should also address specific characteristics that may contribute to parenting stress, as caregiver involvement in interventions carry several benefits. The inclusion of caregivers in the intervention process benefits the family system as a whole and diminishes the time and financial strain associated with several intervention options (Karst and Van Hecke, 2012, p. 259). Lastly, professionals may desire to seek additional training as it relates to the differential diagnosis of intellectual disability and ASD from other common psychiatric diagnoses (e.g., ADHD). This could increase the likelihood children with these co-occurring disorders are connected to appropriate services in a timely manner.

### 4.2. Limitations

Although this study has several improvements over the existing body of literature, the study should be viewed in light of its limitations. First, archival cross-sectional data was used to explore the study hypotheses, resulting in limitations with regard to the sample and measures. It is not possible to make causal statements or to comment on the directionality of the associations identified. Longitudinal studies would be instrumental in doing so. Given the study design, it is difficult to determine which predictor has the strongest association with service complexity as the explanatory power of results is restricted to single indicators. Second, the generalizability of findings is limited to the youth seeking services at mental health care settings across the Province of Ontario.

Third, although there is a variety of medical and psychiatric diagnoses that may be associated with ASD, the full breadth of diagnostic conditions was not examined. Additionally, the three functional levels of ASD (i.e., requiring support, requiring substantial support and requiring very substantial support) were not delineated in this study. Future research in the area may want to expand on the psychiatric and medical diagnoses captured in this study and investigate additional comorbidities across the various functional levels. In the same vein, this research study was concerned with a subset of services, supports and focuses for intervention that may not fully capture youth’s experiences with the health care system. Services administered by other health care professionals that are important in the management of ASD traits among youth are varied and could be further investigated. Fourth, evaluated health status was used in the study to approximate symptom severity. However, admissions to mental health settings are frequently pre-determined by the agency regardless of severity and focuses on the stabilization of symptoms. Therefore, future research may be interested in using more specific proxies of symptom severity when investigating the relationship between severity and mental health service use.

Finally, there are several variables that theoretically may be associated with service complexity that were not captured in this study. For example, ethnicity/race were not included in the present study but have been shown to be related to mental health service use among autistic youth (Taylor and Henninger, 2015; Ryan et al., 2018). Additionally, self-harming and suicidal behaviour, characteristic of a few psychiatric diagnoses included in the current study and linked to autistic youth (Masi et al., 2020, p. 2), were not investigated due to limitations posed by the size of the sample. As this study focused primarily on individual-level factors, future research may want to investigate system-level factors, as they too have a bearing on service utilization trends (Bai et al., 2009). Researchers may want to stratify data on community-level variables (e.g., urbanicity) to tease apart nuances related to geography that have been implicated in youth’s service utilization (Reardon et al., 2017; Brooks et al., 2021). Despite the aforementioned limitations, this research study provides important findings related to co-occurring conditions among autistic youth and highlights areas for future research initiatives.

## 5. Conclusion

Given the treatments for autistic youth can incur high costs for families and society, it is important to gain a comprehensive understanding about service complexity to adequately meet the needs of those who are seeking services and improve access to those who are waiting to be connected to supports. This study addressed a gap in the literature related to service complexity as it relates to predisposing, enabling and need variables. The strengths of this research are obtained from the use of Andersen’s model to a large sample of treatment-seeking youth in Ontario, Canada who were assessed for both psychiatric and medical diagnoses using a comprehensive, standardized data assessment-to-intervention system that has multi-sectoral applicability. Interventions for autistic youth should be mindful of the youth’s age, sex, presence of co-occurring conditions, intellectual disability and their health status. Further, professionals working with this demographic should be considerate of the impact that caregivers have on their child’s access to services and supports. A multidisciplinary approach is very important as several services are accessed for the provision of ASD supports, underscoring the invaluable advantage a standardized tool appropriate for use across service sectors provides. Longitudinal research in the field, using a comprehensive assessment-to-intervention system to support an integrated health information system will be essential to enhance our understanding of, and provide the most effective treatment for, these vulnerable youth.

## Data availability statement

All requests for data should be directed to SS, sstewa24@uwo.ca.

## Ethics statement

The studies involving human participants were reviewed and approved by the Research Ethics Board at Western University. Agencies obtained written informed consent consent from the participants’ legal guardian/next of kin as part of the standard of care. Ethics clearance for secondary analyses of interRAI data gathered by other organizations was obtained from the University of Waterloo (ORE#30173) and Western University (REB#106415).

## Author contributions

SS designed the training, procedures, methodology, and provided the data for the study. VS and NL developed the analytical strategy. VS performed the statistical analysis. All authors contributed to the formulation of the ideas presented in the study and involved in the writing and reviewing of the final manuscript.
